# Supplementary material for: Evaluation of Antioxidant-Rich Mexican Oregano (Lippia graveolens) Infusion and Carvacrol: Impact on Metabolic Activity and Cytotoxicity in Breast Cancer Cell Lines
Source: Nutrients. 2025 Sep 28;17(19):3089. doi: 10.3390/nu17193089 (PMC12525765; doi:10.3390/nu17193089)
Supplement: Supplementary file 1 [file nutrients-17-03089-s001.zip › nutrients-3881451-supplementary.pdf]

Laboratorio Nacional de Identificación y Caracterización Vegetal

TRADITIONAL IDENTIFICATION RESULT

Las Agujas, Zapopan, Jalisco, March 18, 2025

Dra. Trinidad García Iglesias

Centro Universitario de Ciencias de la Salud

PRESENT

Through this letter, we inform you of the results of the taxonomic identification of your samples corresponding to order number SIST-TRA-20250506. The determination was carried out based on morphological characteristics, using dichotomous keys and comparison with specimens deposited in the Luz María Villarreal de Puga Herbarium at the Institute of Botany of the University of Guadalajara (IBUG). Subsequently, the names were verified through consultations of electronic databases available on the internet: Tropicos, International Plant Names Index (IPNI), Plants of the World Online (POWO), and specialized literature. The family classification followed the system proposed by APG IV. The taxonomist in charge of the identification was: Dr. Pablo Carrillo Reyes. The samples were deposited, as a permanent record and safeguard, in the IBUG herbarium.

Sample No.	Scientific name	Family
SIST-TRA-20250506	***Lippia origanoides*** **Kunth**	**Verbenaceae**

It should be noted that this species has been known in Mexico for a long time under the name Lippia graveolens Kunth, which is currently recognized as a synonym of Lippia origanoides Kunth (O'Leary et al., 2012; Calvo & Carnevalli, 2021). This is the species most frequently used as oregano in the American continent (Rzedowski & Calderón de Rzedowski, 2002; Calvo & Carnevalli, 2021).

This document contains information owned by LaniVeg. Any disclosure to third parties must be authorized by the Technical Manager. The original handwritten signatures appearing on the document are safeguarded by the Document Control Manager. Any printed document without the "Controlled Copy" stamp will be considered a "Non-Controlled Copy".

References

Calvo I., L.M. & G. Carnevali F.-C. (2021). ¿Debemos seguir llamando orégano mexicano a *Lippia graveolens* Kunth?: Esclareciendo el nombre del orégano mexicano. *Desde el Herbario CICY 13*: 212-216.

O'Leary, N., Denham, S. S., Salimena, F., & Múlgura, M. E. (2012). Species delimitation in *Lippia* section *Goniostachyum* (Verbenaceae) using the phylogenetic species concept. *Botanical Journal of the Linnean Society*, *170*(2), 197-219.

Rzedowski, J. & G. Calderón de Rzedowski. (2002). Verbenaceae. *Flora del Bajío de Regiones Adyacentes 100*: 1-145.

Sincerely,

Dr. Pablo Carrillo Reyes
Curador Herbario, *"Luz María Villarreal de Puga"* (IBUG),
Instituto de Botánica de la Universidad de Guadalajara

This document contains information owned by LaniVeg. Any disclosure to third parties must be authorized by the Technical Manager. The original handwritten signatures appearing on the document are safeguarded by the Document Control Manager. Any printed document without the "Controlled Copy" stamp will be considered a "Non-Controlled Copy".
